# Ovarian Transcriptomic Analyses in the Urban Human Health Pest, the Western Black Widow Spider

**DOI:** 10.3390/genes11010087

**Published:** 2020-01-12

**Authors:** Lindsay S. Miles, Nadia A. Ayoub, Jessica E. Garb, Robert A. Haney, Brian C. Verrelli

**Affiliations:** 1 Center for Life Sciences Education, Virginia Commonwealth University, Richmond, VA 23284, USA; bverrelli@vcu.edu; 2 Department of Biology, University of Toronto Mississauga, Mississauga, ON L5L 1C6, Canada; 3 Department of Biology, Washington and Lee University, Lexington, VA 24450, USA; ayoubn@wlu.edu; 4 Department of Biological Sciences, University of Massachusetts Lowell, Lowell, MA 01854, USA; Jessica_Garb@uml.edu; 5 Department of Biology, Ball State University, Muncie, IN 47306, USA; rahaney@bsu.edu

**Keywords:** ovary, fecundity, black widow spider, transcriptome, gene expression

## Abstract

Due to their abundance and ability to invade diverse environments, many arthropods have become pests of economic and health concern, especially in urban areas. Transcriptomic analyses of arthropod ovaries have provided insight into life history variation and fecundity, yet there are few studies in spiders despite their diversity within arthropods. Here, we generated a *de novo* ovarian transcriptome from 10 individuals of the western black widow spider (*Latrodectus hesperus*), a human health pest of high abundance in urban areas, to conduct comparative ovarian transcriptomic analyses. Biological processes enriched for metabolism—specifically purine, and thiamine metabolic pathways linked to oocyte development—were significantly abundant in *L. hesperus*. Functional and pathway annotations revealed overlap among diverse arachnid ovarian transcriptomes for highly-conserved genes and those linked to fecundity, such as oocyte maturation in vitellogenin and vitelline membrane outer layer proteins, hormones, and hormone receptors required for ovary development, and regulation of fertility-related genes. Comparative studies across arachnids are greatly needed to understand the evolutionary similarities of the spider ovary, and here, the identification of ovarian proteins in *L. hesperus* provides potential for understanding how increased fecundity is linked to the success of this urban pest.

## 1. Introduction

Arthropods have long been used as models in studying life history variation and evolutionary adaptation [1,2,3]. Arguably one of the most important life history traits is fecundity, as it is closely tied to fitness, and studies of fecundity and population abundance highlight the arthropod literature [4,5,6]. Arthropods also dominate the literature as invasive and pest species as a result of their high fecundity and ability to rapidly adapt to different environments, which include the urban and agricultural areas that humans have created. A few human pest examples are the common flour beetle, due to their close affinity with grain storage [7,8,9]; aphids, of which over 80 global species destroy crops and ornamentals [10]; and termites that feed on wood in homes [11,12,13]. However, from an ecological and evolutionary perspective, some of the more interesting examples come from those arthropod pest species that are anthropogenic obligates to the extent that they have become threats to human health. For example, cockroaches, bedbugs, and even mosquito species [14,15,16] have adapted to these newly-formed environments, and their abundance implies they thrive within them. In this context, there is a great need to characterize the genotypic and phenotypic responses associated with pest species’ adaptation to human environments to prioritize management and health concerns [17].

With genomic and transcriptomic tools available for any species, comparative and functional analyses of gene expression can be used to investigate fitness-related traits in non-model arthropod pest species in human-altered environments. Ovaries fulfill several important functions tied to oocyte development, hormone secretion, and fertilization, and thus, transcriptomic analyses of these tissues can shed light onto factors driving fecundity. Adrian and Comeron [18] analyzed ovary transcript profiles of early- and late-meiosis development stages in *Drosophila melanogaster* and showed maternal effects differ across developmental stages. In doing so, they identified new transcripts that likely map to regions of heterochromatin, which is surprising given the very deep genomic sequencing of this model organism. Yang et al. [19] examined the ovary transcriptome in *Portunus trituberculatus*, the Japanese blue crab and the most widely fished species of crab in the world, and found significant variation in gene expression across sexual maturation stages that can help increase population size for this economically-relevant species. Finally, Uengwetwanita et al. [20] examined the ovary transcriptome in *Penaeus monodon*—the black tiger shrimp, and again a species of economic importance—to show that although new genes were identified, gene regulation followed similar pathways identified in vertebrates. These studies highlight how comparative transcriptome analyses reveal both unique and similar patterns of gene expression that can be used to understand the ecology and evolution of fecundity in arthropods of interest to humans.

The western black widow spider (*Latrodectus hesperus*) has become a pest species of human medical relevance due to its potent venom and unique characteristics as an urban adaptor [21,22,23,24]. Specifically, urban *L. hesperus* show higher fecundity and more dense aggregations than non-urban spiders [25,26,27]. Our previous sampling of thousands of genomewide single nucleotide polymorphisms (SNPs) from 21 urban and non-urban southwestern U.S. locales found higher genetic diversity within urban areas and higher gene flow among urban areas [28], a result that contrasts with the standard hypothesis associated with urbanization [29]. Additionally, our ‘popgraph’ network analysis identified specific urban ‘hubs’ that contribute more to *L. hesperus* population expansion and gene flow than others, and has implications for pest management [30]. These ecological and genetic patterns raise the question of what phenotypic variation exists for the genes and pathways associated with *L. hesperus* fecundity, not only between urban environments but also in comparison to other arachnid species. However, despite their unique phylogenetic and taxonomic diversity, there is a pronounced paucity of transcriptome information available overall for spiders, and these studies typically focus on venom and silk [31,32,33,34,35,36,37,38]. In fact, only two ovary studies have examined the biological and functional dynamics of transcript variation associated with fecundity [39,40].

Although tissue-specific transcriptome data exist for *L. hesperus*, there are no ovary-specific expression analyses and no annotated genome available, without which we struggle to understand factors underlying fecundity variation that drives population expansion in this urban pest. Here, we provide comparative ovarian transcriptomic analyses of the western black widow spider from tissue-specific RNA-seq data collected from multiple individuals from a population sample in the southwestern United States. Our overall objective is to construct a *de novo* ovarian transcriptome for *L. hesperus* to compare with other spiders and arthropods in identifying genes and pathways associated with ovarian expression and function. Addressing this objective will contribute to our understanding of the evolutionary diversity of arthropod ovaries in general, and will be specifically important to our understanding of the functional variation associated with fecundity in the western black widow spider as an urban pest model of human health concern.

## 2. Materials and Methods

### 2.1. Sample Collection and RNA Extraction

We collected 10 *L. hesperus* adult female spiders in June, 2017, during their breeding season, from an urban locale within the Phoenix (AZ, USA) city limits. Specifically, individuals were collected from a single area of infestation (GPS coordinates: 33.618972, −112.065231). As is typical of this species within urban areas (Miles et al., 2018a, b; [28,30]), individuals were found within their webs at high densities covering brick walls and rocky outcrops of artificial, man-made surfaces facing a major city street. Ovaries from each individual were dissected in 150 mM sodium chloride, 15 mM sodium citrate using forceps and a dissection microscope, and snap frozen in liquid nitrogen. Total RNA was initially isolated from the ovary tissue using Trizol (Invitrogen, Waltham, MA, USA) and chloroform extraction, then further purified using the RNeasy kit (Qiagen, Hilden, Germany), and any contaminating DNA was removed with on-column DNase treatment (Qiagen). The concentration and purity of the extracted RNA was quantified using an Agilent 2100 Bioanalyzer (Santa Clara, CA, USA).

### 2.2. Illumina Library Prep and Sequencing

The cDNA libraries were generated with the TruSeq RNA Sample Preparation Kit (Illumina, San Diego, CA, USA), followed by paired-end, 150 bp sequencing in one lane of HiSeq 4000 (Illumina). Reads were cleaned using *Trim Galore!* v.0.3.7 (http://www.bioinformatics.babraham.ac.uk/projects/trim_galore) with FastQC v.0.11.2 (http://www.bioinformatics.babraham.ac.uk/projects/fastqc/) that removed Illumina adaptors and low-quality reads.

### 2.3. De Novo Assembly of Ovary Transcriptome

There is currently no complete genome available for *L hesperus* or any closely related species. For this reason, and because our initial inspection of the data revealed very high transcript abundance and coverage compared to former transcriptome datasets of *L hesperus*, we took this opportunity to generate a *de novo* ovarian transcriptome. The clean reads were pooled for *de novo* assembly using default parameters in Trinity v.2.5.1 [41,42]. Benchmarking Universal Single-Copy Orthologs (BUSCO v3) was used with default parameters to assess the completeness of the transcriptome assembly using the ‘eukaryota odb9’ database, which contains 303 highly conserved genes [43,44]. Salmon v.1.55 [45] was used to quantify transcript abundance in the ovary transcriptome using the quasi-mapping algorithm.

### 2.4. Functional Annotation

Our methods are aligned with our objective to identify genes and pathways expressed in the ovary and compare these with other ovarian data in arachnids. At this time, we did not focus on analyses that attempt to identify those transcripts specific to or enriched in the ovary compared to other tissues. Instead, similar to other arthropod studies, we desired to first generate a complete dataset of genes and pathways expressed in the ovary. Although we expect this current approach will reveal a number of genes that are universally expressed across tissues (i.e., ‘housekeeping’ genes), this approach is vital for our current comparative analyses, as well as for our follow-up studies to investigate genes and pathways integral to western black widow spider ovarian evolution in urban areas. In fact, we expect genes involved in urban adaptation are linked to fecundity and ovarian pathways, but likely are not genes unique to the ovary. Finally, we also did not attempt to identify ‘unique transcripts’. In doing so, it would reduce the number of redundancies in the data, but it also will remove closely-related genes/orthologs (i.e., recent duplication) that are of interest down the road. Thus, here, we outline methods focused on analyses of transcript data initially interpreted as isoforms for an exhaustive dataset at this stage, and we evaluate our results in light of this approach in the Discussion.

The transcript isoforms were mapped to the NCBI nt database using blastn [46] in the Blast v2.7.1+ package (http://blast.ncbi.nlm.nih.gov) with *e*-value 1 (e-20), max_target_seq 1 and outfmt 6 parameters changed. Next, the transcript isoforms were mapped against the UniProt database to identify functional proteins using Blastx. Finally, excluding isoforms that had not been matched to UniProt, we mapped the remaining isoforms against the only two previously published arachnid ovary-specific transcriptomes, the Spanish funnel-web spider, *Macrothele calpeiana* [39], and the common house spider, *Parasteatoda tepidariorum* [40], to identify overlapping arachnid ovary genes.

To determine the transcripts with nearly full-length proteins and their corresponding UniProt BLAST hits, we first used Blast v2.7.1+ to determine the best matching proteins with the UniProt database, then we used a Trinity Perl script to extract the transcripts with >80% coverage of the UniProt proteins. Next, we extracted these transcripts and their corresponding UniProt BLAST hits to be used in the Gene Ontology (GO) analysis to identify genes in the transcriptome with high transcript abundance for the ‘biological process’, ‘cellular component’, and ‘molecular function’ categories in the ovary. The program PANTHER v.14.1 [47] was used to perform the GO analysis. We generated an annotation report using Trinotate v.3.2.0 [48], then uploaded the annotation results to Blast2GO [49] to perform the Kyoto Encyclopedia of Genes and Genomes (KEGG) analyses, which identify potential functional pathways in the ovary.

## 3. Results

### 3.1. Sequencing and De Novo Assembly

For each of the 10 *L. hesperus* libraries, 35M-63M raw sequence reads were generated, and 98% of clean reads were retained after pre-processing (e.g., adaptor removal, quality trimming, ‘N’ removal). The assembly of transcripts resulted in a total of 690,256 unique transcript isoforms. The BUSCO analysis of the assembled ovary transcriptome resulted in 96% completeness, with 28% complete and single copy (N = 85 of 303 highly conserved genes), 68% complete and duplicate copy (N = 207 of 303), and 4% fragmented (N = 11 of 303). There are 5842 proteins that are represented by nearly full-length transcripts, having >80% alignment coverage and 4021 proteins that are covered by more than 90% of their protein lengths. Using a standard minimum threshold of five ‘transcripts per million transcripts’ (TPM), we found 64,580 transcript isoforms with this minimum threshold of expression in the ovary, and which were used to further characterize genes in our GO and KEGG analyses.

### 3.2. Functional Annotation and Classification

**Figure 1:**
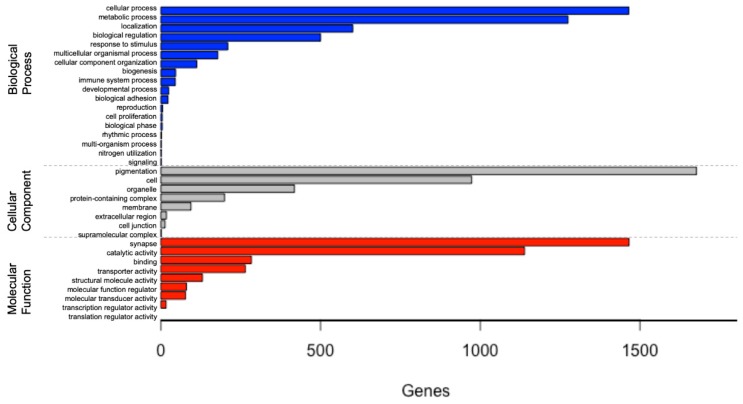
Gene Ontology (GO) analysis of the *Latrodectus hesperus* ovarian transcriptome according to the identified transcripts involvement in biological process, cellular component, and molecular function categories.

From the 64,580 transcript isoforms (noted above), there were 37,387 transcripts for the *L. hesperus* ovary that were assigned GO terms, with each of these transcripts assigned to one or more GO terms in the biological process, cellular component, and molecular function categories (Figure 1). This analysis finds that the GO terms in the *L. hesperus* ovary transcriptome are highly abundant for ‘biological processes’. Within this category, there were 4495 hits with cellular (33%) and metabolic (28%) processes as the most abundant (Supplementary Materials Figure S1, Table S1). For the cellular component category, there were 3392 hits with cell (49%) and organelle (29%) components as the most abundant. Finally, in the molecular function category, there were 3455 hits with catalytic activity (42%) and binding (33%) function as the most abundant.

**Figure 2:**
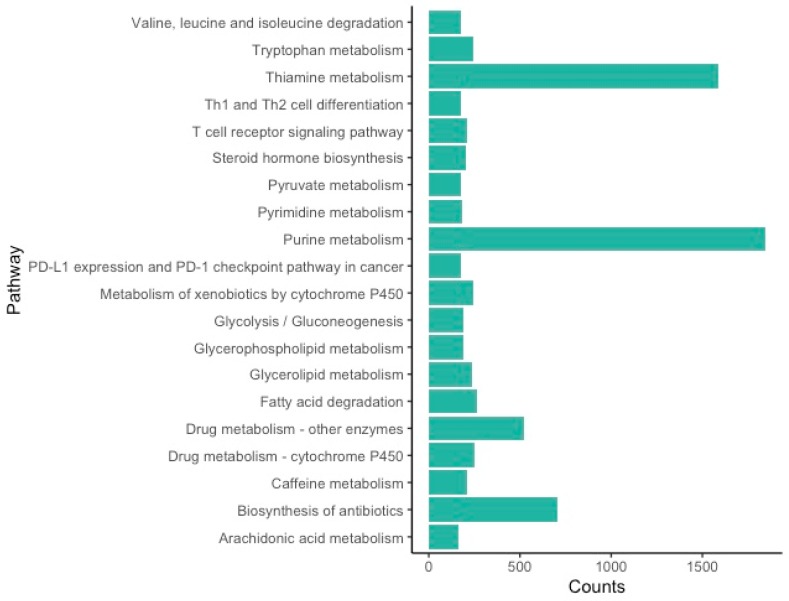
Kyoto Encyclopedia of Genes and Genomes (KEGG) Top 20 pathways for the *L. hesperus* ovarian transcriptome.

The KEGG orthology (KO) assignments resulted in an annotation of 160 pathways that correspond to 14,878 transcripts in the ovary transcriptome (Table S2). The most abundant pathways are related to metabolism (Figure 2), with the two most representative pathways being purine metabolism and thiamine metabolism (Supplementary Materials Figure S2). Purine metabolism plays a role in cell growth or division, with expression in the ovary tied to the cell proliferation of oocytes [50,51]. Thiamine metabolism plays a role in the activity of key enzymes in cellular metabolism and has been linked to the meiotic maturation of oocytes [52,53].

### 3.3. Spider Ovary Genes of Interest

**Figure 3:**
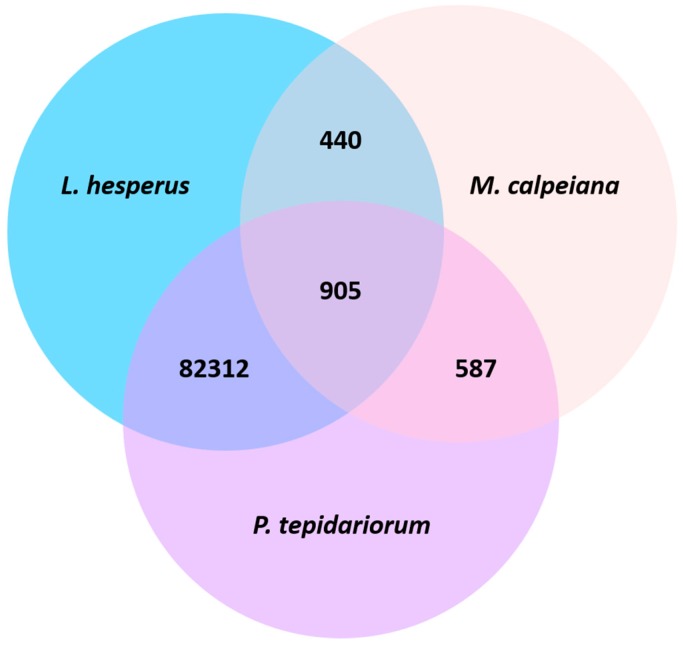
Transcripts overlapping in the ovarian transcriptomes among the three arachnids *L. hesperus*, *Macrothele calpeia na*, and *Parasteatoda tepidariorum*.

The UniProt BLAST identified 551 transcripts whose highest match was listed as an arthropod-annotated gene. Within these 551 top hits, 28 aligned to arachnid genes and 7 of these arachnid genes were associated with α-latrotoxins, which are toxin components found in *Latrodectus* spider venom [54]. Additionally, our BLAST search against *M. calpeiana* and *P. tepidariorum* ovarian transcriptomes resulted in an overlap of 905 transcripts among all three species, 440 shared between *L. hesperus* and *M. calpeiana*, and 82,310 shared between *L. hesperus* and *P. tepidariorum* (Figure 3). However, our *L. hesperus* transcriptome had an additional 80,184 transcripts that match to UniProt genes, but which did not match to transcripts in the other two arachnid ovary transcriptomes.

Although the vast majority of overlapping transcripts are of ‘housekeeping’ genes found in all cell types, Table 1 shows 15 genes identified in our transcriptome that overlapped with the 19 previously-identified fecundity-related genes in arthropods [55,56,57,58,59,60,61,62,63,64,65]. These genes include vitellogenin and vitelline membrane outer layer protein, which are involved in oocyte development [66]. Several hormones and hormone receptors are required for development in the ovary including 3-β-hydroxysteroid dehydrogenase, mandibular organ-inhibiting hormone, lutropin-choriogonadotropic hormone receptor, follicle-stimulating hormone receptor, and estrogen [67]. Lastly, genes that regulate vitellogenin and other fertility-related genes in the ovary include zinc-finger protein, phosphoglycerate kinase, carboxylesterase, protein geranylgeranyl transferase, SRY related HMG-Box-11, C-terminal-binding protein, fizzy, sex-lethal, serine-threonine kinase receptor, integrin protein, rab guanosine diphosphate/guanosine triphosphate exchange factor, and maternal protein pumilio [65]. Interestingly, only seven of these 15 fecundity-related genes (3 β-hydroxysteroid dehydrogenase, estrogen, zinc-finger protein, phosphoglycerate kinase, protein geranylgeranyl transferase, SRY related HMG-Box-11, C-terminal-binding protein) overlapped with the *P. tepidariorum* ovarian transcriptome, whereas none of them overlapped with *M. calpeiana*.

## 4. Discussion

The overall objective of this study was to construct a *de novo* ovarian transcriptome for *L. hesperus* to compare with other spiders and arthropods in identifying genes and pathways associated with ovarian expression and function. Because no genome is yet available for *L. hesperus*, a *de novo* assembly of the transcriptome was generated from a large dataset with transcript coverage. Specifically, our annotation rate was 54.5%, which is similar to other arthropod studies, even though these previous studies have an available genome. For example, the annotation rate is ~50% for several arthropods including *Antheraea pernyi* (50.8%), *Helicoverpa armigera* (50.8%), *Helicoverpa assulta* (54.0%), *Spodoptera frugiperda* (51.1%), and *Athetis lepigone* (41.5%) [68,69,70]. With only two spider ovarian transcriptomes assembled, *L. hesperus* has a significantly higher annotation rate than *M. calpeiana* (29.4%) [39] and *P. tepidariorum* (19.7%) [40]. These results reflect a successful *de novo* assembly and provide for comparisons with ovarian transcriptomes of other spiders.

### 4.1. Western Black Widow Spider Ovarian Gene and Pathway Categories

The main function of the ovary in spiders, and arthropods in general, is to develop mature oocytes and produce hormones needed for female reproduction. Thus, the identification of genes and pathways with transcripts of high abundance tied to biological processes involved in metabolism and cellular proliferation in the ovary is expected, and is a pattern that is consistent with other arthropod transcriptome studies [39,71,72,73]. Sub-groups of cellular processes and metabolic processes have the first and the second highest transcript count, respectively, for GO term annotations (Figure 1), which is consistent with other arthropod studies of ovarian transcriptomes.

While metabolic processes in general have been found as top KEGG pathways in other arthropods [39,72,74,75], this is the first study to identify purine and thiamine metabolism as the top pathways in an arthropod ovarian transcriptome. Purine metabolism has been well-documented as a key factor in cell growth and division during oocyte proliferation in mice [50,51]. Purine pathways are important in both follicular fluid and serum surrounding oocytes, which aid in the development of oocytes [76]. If purine metabolism is reduced, oocyte development is suppressed [76,77,78]. Purine metabolism is linked to oocyte quality, which can lead to lower fertility when there are changes to this metabolic pathway [51,76]. Similarly, thiamine metabolism has been identified as part of cellular metabolism and meiotic maturation of oocytes in mice [52,53]. Arthropods that are unable to sequester thiamine in their diets, such as tsetse flies and ticks, have reduced fertility when their symbiont—typically a bacteria that produces thiamine—is removed [79]. The reduced fertility in these arthropods is likely caused by vitamin B deficiencies that can inhibit meiotic maturation of oocytes [53]. While this study suggests these pathways may be major factors in ovarian development, investigating these patterns (such as vitamin deficiencies) in other closely-related spiders and within *L. hesperus* will determine whether they play a unique role in this species.

### 4.2. Species Comparisons of Spider Ovarian Transcriptomes

Although only two other spider ovary-specific transcriptome datasets are available, there are already a few interesting lessons to be learned here. We find that there is significantly more overlap among *L. hesperus* and *P. tepidariorum* than between *L. hesperus* and *M. calpeiana*. In fact, there is more overlap among all three transcriptomes than there is shared between *L. hesperus* and *M. calpeiana*. There are several potential explanations for these patterns, the first being the different datasets. Similar to our transcriptome, the *P. tepidariorum* data are generated from the ovaries of adult female spiders, and these data include all raw sequences, i.e., sequenced transcripts that blast to known and unknown genes [40]. The *M. calpeiana* data are also generated from the ovaries of adult female spiders, but these data are the result of a ‘subtractive’ approach, i.e., an attempt by the authors to enrich for tissue-specific transcripts at various steps [39]. As noted previously, this approach was specifically not taken by us to help guard against a bias towards ovary-specific transcripts, which may have less overlap with other spider and arthropod transcriptomes, as we speculate here.

Given the approach by Frias-Lopez [39], the overlap between *L. hesperus* and *M. calpeiana* may more reflect ‘ovary-specific’ transcripts in contrast to the *L. hesperus* and *P. tepidariorum* overlap. However, inspection of this overlap between *L. hesperus* and *M. calpeiana* finds the vast majority are standard housekeeping genes. In addition, we might expect the majority of genes in common with ovary development and oocyte production should be shared among the three species. However, over 98% of the three-species overlapping transcripts also map to housekeeping genes with >90% identity, and none of the arthropod fecundity-related genes exist in this three-species overlap. Instead, of the 19 fecundity-related genes, only 7 are shared between *L. hesperus* and *P. tepidariorum* and none are shared between *L. hesperus* and *M. calpeiana*. One consideration is that these genes are expressed differentially due to evolutionary changes in oocyte development. It is possible that if gene expression across oocyte developmental stages is examined (as in Drosophila) [18], that we find that these genes conserved in arthropods have diverse roles in egg maturation even among spiders.

Another explanation for these patterns in overlap among spider species is that *L. hesperus* and *P. tepidariorum* (from the Theridiidae family), are more phylogenetically related [80,81], whereas, the divergence time of *M. calpeiana* (Macrothelidae family) from the other two is estimated at over 300 million years [82]. Thus, given this significantly higher divergence, we expect that overlap among the three will largely reflect highly evolutionarily conserved genes, which is in fact what we see. This result alone does not imply that these housekeeping transcripts do not have ‘ovary-specific’ functions, especially given the curation of the *M. calpeiana* dataset; however, more analyses would be needed to test this hypothesis. For example, we may speculate that a proportion of the transcripts do not overlap because they have sufficiently diverged due to positive selection as a response to ovary-specific adaptation—a hypothesis we will address with molecular evolutionary analyses of these genes within and between species. These results make the obvious argument that more ovarian transcriptomes are needed from closely-related spiders to determine whether these annotated transcripts with no overlap are truly species-specific.

### 4.3. Western Black Widow Spider Genes Associated with Fecundity

Our analyses of the *L. hesperus* transcriptome identified 15 of 19 genes relevant for fecundity and the process of oocyte growth previously noted in arthropod ovaries [55,56,57,58,59,60,61,62,63,64,65]. These studies had identified serine-threonine kinase receptor, integrin protein, rab guanosine diphosphate/guanosine triphosphate exchange factor, and maternal protein pumillo as involved in arthropod fertility and vitellogenin regulation [65]. However, these four genes were not identified in our transcriptome. As noted previously, the lack of these transcripts in our dataset may less reflect the loss of the genes in *L. hesperus*, but instead that there is some temporal stage of expression or population variance. For example, there were several vitellogenin regulation genes that we did identify, and the lack of these four genes (listed above) in our transcriptome may indicate that not all of them are required to be expressed during oocyte development. Indeed, the hormonal control of vitellogenesis in spiders is not well understood, and there is a paucity of data on the hormones involved [83]. Finally, as previously noted, it is also possible that these genes were not identified because they have sufficiently diverged due to positive selection compared to other arthropod ovarian genes. There are a number of transcripts in *L. hesperus* that were annotated but do not overlap with the two species, and thus, our planned evolutionary analyses of transcripts initially annotated as ‘species-specific’ can address this hypothesis.

For each of the fecundity-related genes identified in *L. hesperus*, we identified evidence consistent with alternative splicing (Table 1). In fact, the most abundant represented gene is the zinc-finger protein, with 315,938 transcripts aligned. The zinc-finger protein is an abundant transcription factor in many transcriptomes, and its primary role is to regulate gene expression [84,85]. One of the most ubiquitous genes present in arthropod ovaries that is likely regulated by these zinc-finger transcription factors is vitellogenin, which is used in the process of oocyte development [66]. Our results indicate that these genes are also undergoing alternative splicing, as we find 184 transcripts of the vitellogenin gene, a pattern found in arthropods, and more specifically in other arachnids [19,86,87,88,89]. Vitellogenesis involves the synthesis and accumulation of proteins for eggs to develop [66]. Mated female spiders have ovaries in vitellogenesis and post-vitellogenesis phases, which would produce significant levels of vitellogenin proteins [90,91,92,93]. As our current study includes only gravid females, it would be of interest to examine different female stages (i.e., unmated, multiple matings, etc.) to detect potential adaptive expression variation in fecundity.

### 4.4. Fecundity and Human Pest Adaptation to Urban Environments

The western black widow spider is an example of a human pest that has thrived due to human-facilitated movement [28,29,30], and our identification of ovarian transcriptome patterns here has implications for its adaptation to urban environments. One interesting result was the identification of many isoforms associated with α-latrotoxin, which is a member of a family of highly-expressed neurotoxins found in *Latrodectus* spider venom that is specific to vertebrates [54]. Haney et al. [40] also identified these transcripts in their ovarian transcriptome for *P. tepidariorum*, and they were found here in the overlap with *L. hesperus*, but not between *L. hesperus* and *M. calpeiana*. Toxin-related transcripts have been identified in *L. tredecimguttatus* eggs, to which the authors speculated the eggs have their own toxic mechanism [94]. The ovaries collected in this study were from gravid females, with visible oocytes, and therefore these toxins may be produced in the yolk. Our sample was collected from an urban geographic area of Phoenix, AZ, and urban spiders have higher fecundity than non-urban spiders [25,26,27]. Thus, this additional toxin in the yolk may play a role in higher fitness of the urban egg sacs relative to non-urban ones. Future studies can test this hypothesis by measuring differential gene expression of these latrotoxins in ovaries between urban and non-urban populations to elucidate if this pattern reflects a unique response to the urban environment.

Although vitellogenin transcripts in the ovarian transcriptome are expected for an arthropod, it is unclear whether we may expect high expression of these genes to be associated with increased fecundity. Previous studies have suggested that urban *L. hesperus* produce significantly lower ‘quality’ eggs compared to non-urban spiders [25,26,95]; however, urban spiders have been shown to produce significantly more eggs per egg sac. Thus, it may be the case that selection in urban areas produces smaller, but more eggs compared to non-urban areas as a result of reduced interspecific competition and increased intraspecific competition [25,26,95]. In fact, the pattern seen here of genes linked to cell proliferation and vitellogenin and its regulation, could be consistent with higher fecundity in urban areas being adaptive. Future work can target these genes of interest between urban and non-urban populations to tease apart quality and quantity differences in reproductive success.

Much of what we have learned about how urban adapter pests perform and thrive in the urban environment is due to contrasts between populations, species, and phenotypes. This current study highlights the value and gaps in tissue-specific and comparative species analyses. For example, while we need more species transcriptomes to resolve whether annotated transcripts are species-specific, we also need more tissue-specific transcriptomes to determine whether genes and pathways reflect tissue-specific function. In fact, other than fecundity, there are other urban adapter phenotypes that co-evolve and contribute to urban adaptation. For example, previous studies have identified prey differences between urban and non-urban populations [27,96] that likely affect gene expression in food metabolism, venom production, and prey-capturing silk. Therefore, the analysis of transcriptome patterns across multiple tissues associated with these phenotypes across both urban and non-urban populations would reveal the genetic architecture involved in the adaptation of pest species, such as the western black widow spider, to human-altered environments.

## Figures and Tables

**Table 1:** Arthropod fecundity-related genes present in the *L. hesperus* ovarian transcriptome.

Gene.	Function	Count *
Vitellogenin	oocyte development	181
Vitelline membrane outer layer protein	oocyte development	174
Estrogen	ovary development	3397
3 beta-hydroxysteroid dehydrogenase (3-β-HSD)	ovary development	405
Mandibular organ-inhibiting hormone (MOIH)	ovary development	100
Lutropin-choriogonadotropic hormone receptor (LSHR)	ovary development	30
Follicle-stimulating hormone receptor (FSHR)	ovary development	5
Zinc-Finger protein (ZFP)	vitellogenin regulation	315938
phosphoglycerate kinase	vitellogenin regulation	5424
carboxylesterase	vitellogenin regulation	2610
C-terminal-binding protein (CtBP)	vitellogenin regulation	584
protein geranylgeranyl transferase	vitellogenin regulation	297
fizzy (fzy)	vitellogenin regulation	244
SRY related HMG-Box-11 (SOX-11)	vitellogenin regulation	145
Sex-lethal (Sxl)	vitellogenin regulation	5

* The number of reads identified as the given name.

## Supplementary Materials

The following are available online: https://www.mdpi.com/2073-4425/11/1/87/s1, Table S1: GO terms and the number of *L. hesperus* ovarian transcripts per GO category; Table S2: KEGG pathway annotations for *L. hesperus* ovarian transcriptome; Figure S1: Gene Ontology (GO) level distribution of transcripts in the *L. hesperus* ovarian transcriptome, Figure S2: Purine and thiamine metabolism pathways in the *L. hesperus* ovarian transcriptome.
